# Lysine-374 lactylation of TFRC is essential for TFRC-mediated kidney tubular ferroptosis in diabetic kidney disease

**DOI:** 10.1016/j.jbc.2026.113429

**Published:** 2026-08-10

**Authors:** Xiaomai Liu, Runzhu Yuan, Wenya Zhu, Jiaqing Xiang, Guangyan Yang, Lixing Li, Yanchun Li, Lin Kang, Shu Yang, Zhen Liang

**Affiliations:** 1 Department of Geriatrics, The First Affiliated Hospital (Shenzhen People's Hospital), School of Medicine, Southern University of Science and Technology, Shenzhen, China; 2 Guangdong Provincial Clinical Research Center for Geriatrics, Shenzhen Clinical Research Center for Geriatrics, The First Affiliated Hospital, Southern University of Science and Technology (Shenzhen People’s Hospital), Shenzhen, Guangdong, China; 3 Department of Geriatrics, Peking University Shenzhen Hospital, Shenzhen, Guangdong, China; 4 Institute of Health Medicine, Southern University of Science and Technology, Shenzhen, Guangdong, China

**Keywords:** DKD, TFRC, lactylation, ferroptosis, p300

## Abstract

Diabetic kidney disease (DKD), a leading cause of end-stage kidney disease, lactate overload and ferroptotic kidney injury, yet the mechanistic link between lactylation and ferroptosis remains undefined. Using lactylome profiling of *db/db* mouse kidneys, we identified transferrin receptor (TFRC) as the key ferroptosis regulator modified by lactylation. Here we explore the pathogenic role of TFRC lactylation in DKD progression. We observed significantly elevated lactate levels and specific lactylation of TFRC at lysine 374 (K374) in the kidneys of *db/db* mice and lactate-stimulated HK2 cells. This modification was strongly correlated with increased iron accumulation, lipid peroxidation, and kidney fibrosis. Mechanistically, through screening of canonical lactylation-associated enzymes, we identified histone acetyltransferase p300 as the major enzyme catalyzing TFRC K374 lactylation. Utilizing a non-lactylatable TFRC mutant (K374R), we demonstrated that preventing this modification represses lactate-induced ferroptosis and profibrotic signaling both *in vitro* and *in vivo*. Virtual screening of an endogenous compound library identified lysicamine as a p300 inhibitor, which effectively reduced TFRC lactylation and mitigated kidney injury and fibrosis. Collectively, these findings uncover a critical “lactate-p300-TFRC lactylation” axis that drives ferroptosis in DKD, suggesting that targeting TFRC K374 lactylation offers a novel therapeutic strategy for preserving kidney injury in diabetic patients.

Diabetes is a chronic metabolic disorder that affects most major organs of the body (1). In 2019, about 463 million people globally had diabetes. According to relevant statistical projections, the number of people with diabetes is expected to rise to 700 million by 2045 (2). Proximately 30% to 40% of individuals with diabetes will develop diabetic kidney disease (DKD) during their lifetime (3, 4). DKD is a leading cause of end-stage kidney disease (ESKD), posing a significant threat to human health (5, 6). Our limited grasp of DKD's development hinders the creation of effective therapies.

Under normal physiological conditions, kidney cells mainly depend on mitochondrial oxidative metabolism to generate energy (7, 8, 9, 10, 11). However, in DKD, hyperglycemia inhibits mitochondrial oxidative phosphorylation (12, 13). Instead, kidney cells rely on glycolytic metabolism, exhibiting a Warburg effect similar to cancer cells. While this pathway rapidly meets specific cells' energy demands, it also increases lactate production and accumulation (14, 15, 16). Lactate concentration is closely linked to lactylation modification, with both components forming a cascade amplification effect through a “metabolism-modification” feedback loop (17, 18). Lactylation modification regulates proteins across several inflammatory signaling pathways. Elevated lactate levels induce H3K14 lactylation, activating the transcription factor KLF5. Activated KLF5 binds to and suppresses the expression of epithelial cell adhesion protein E-cadherin. This accelerates the epithelial–mesenchymal transition (EMT) process in DKD, promoting kidney fibrosis (14). TRIM65 is lactoylated at K206 by lactate, which inactivates it and links lactate-driven tubular injury to ferroptosis and glycolysis in DKD progression (19). Collectively, these findings highlight a pivotal “metabolic-reprogramming-lactylation” axis in DKD, wherein glycolytic shift and lactate accumulation drive profibrotic and pro-ferroptotic responses through protein lactylation, thereby accelerating disease progression.

DKD is closely linked to ferroptosis (10). Hyperglycemia in DKD can disrupts normal iron metabolism, causing kidney cells to absorb excessive iron. This iron accumulation creates conditions that can trigger ferroptosis (20, 21). As a key gateway for cellular iron import, transferrin receptor (TFRC) plays a crucial role in ferroptosis (22, 23). For instance, when levels of TFRC rise, cells absorb more Fe^3+^, which is then reduced to Fe^2+^. This Fe^2+^ drives the Fenton reaction, producing hydroxyl radicals. These radicals attack the polyunsaturated fatty acids (PUFAs) in cell membranes, causing lipid peroxidation and cell death (24, 25). Additionally, Fe^2+^ also binds to GSH, depleting GSH reserves and reducing GPX4 activity. This leads to lipid peroxide accumulation within cells, further promoting ferroptosis (26, 27). Although the role of TFRC in triggering ferroptosis in DKD is well-established, research on its lactylation modification is still limited. It remains unclear whether TFRC undergoes lactylation and what the functional impact of such a modification might be.

In this study, using global lactylation proteomic profiling, we identified a critical role for TFRC K374 lactylation in the pathogenesis of diabetic kidney disease. We found that in *db/db* mice, elevated lactate levels drive the lactylation of TFRC at K374, contributing to kidney fibrosis and ferroptosis. By utilizing TFRC K374R mutants and p300 inhibitor lysicamine (LCA), we demonstrated that blocking this lactylation significantly reduces kidney injury and fibrosis in DKD mice. Furthermore, our research identified p300 as the primary enzyme catalyzing TFRC K374 lactylation and showed that LCA effectively protects kidneys by inhibiting p300 and reducing TFRC lactylation. These findings highlight the potential of targeting TFRC K374 lactylation as therapeutic strategies for DKD.

## Results

### Elevated kidney lactate levels and TFRC K376 lactylation in DKD mice

**Figure 1:**
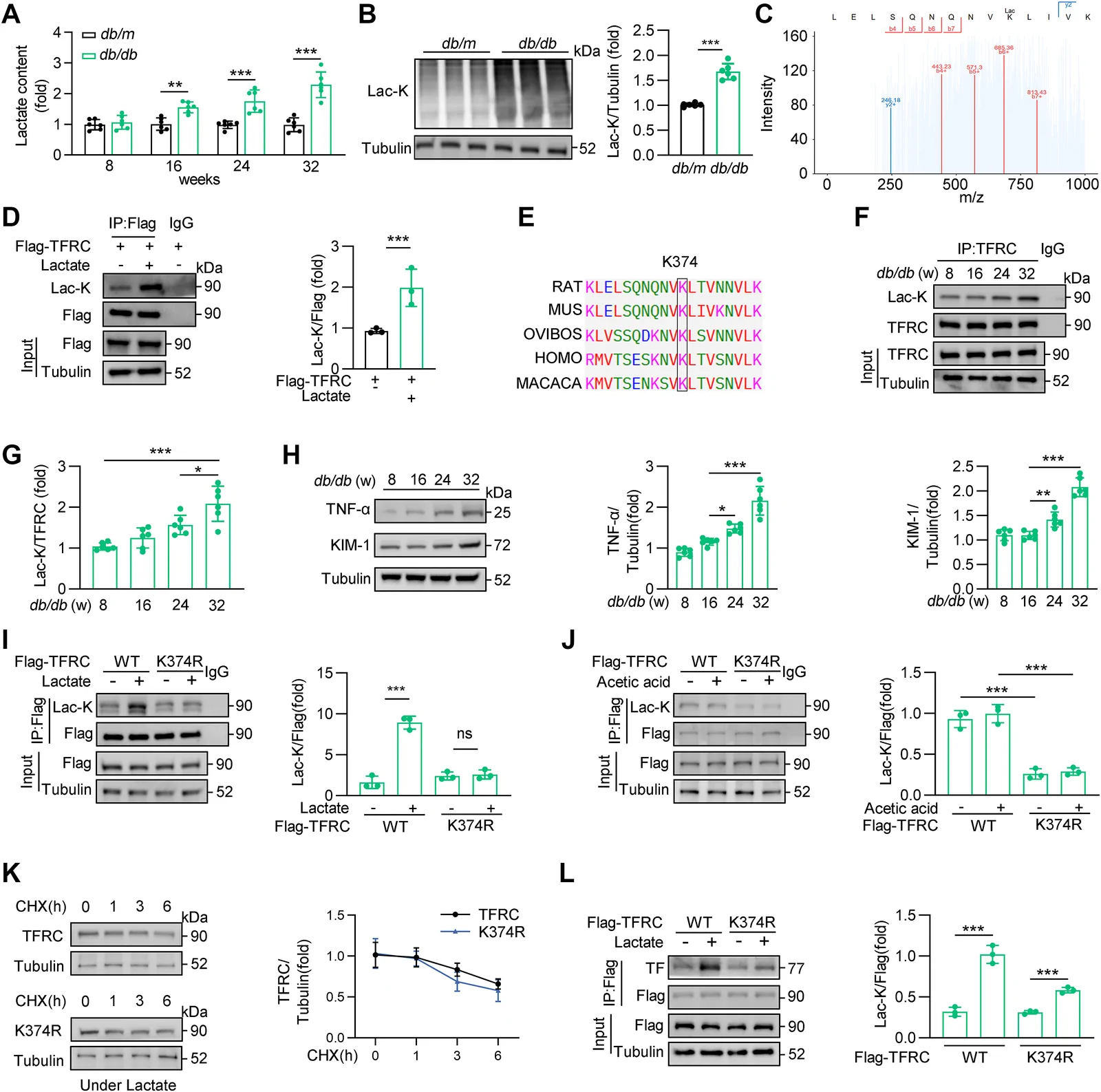
**In DKD mice, lactate levels rise and trigger lactylation modification at TFRC K376.***A*, kidney lactate levels were measured in *db/m* mice and *db/db* mice at different ages (*n* = 6). *B*, kidney cell lysates were analyzed by Western blotting using anti-Lac-K antibody in *db/m* and *db/db* mice. Quantitative results are shown in the figure on the right (*n* = 6). *C*, Liquid Chromatography-Mass Spectrometry (LC-MS) Analysis of lactylation modification Sites in *db/db* Mouse Kidneys. *D*, WT HK2 cells were transfected with Ad Flag-TFRC with or without 10 mM lactate. Co-immunoprecipitation (CoIP) was performed on total cell lysates using anti-Flag antibody, followed by Western blot analysis. Quantitative results are shown in the *right panel* (*n* = 3). *E*, Conservation of TFRC K374 across different species. *F*, CoIP was performed using anti-TFRC antibodies on kidney cell lysates from *db/db* mice at different ages, followed by Western blot analysis. *G*, quantification of TFRC lactylation levels in db/db mice at different ages (*n* = 6). *H*, kidney cell lysates were extracted and subjected to Western blotting using designated antibodies in *db/db* mice at different ages. Quantitative results are shown in the figure on the right (*n* = 6). *I*, HK2 cells were transfected with Ad Flag-TFRC WT or Ad Flag-TFRC K374R, with or without lactate. CoIP was performed on total cell lysates using anti-Flag antibody, followed by Western blot analysis with designated antibodies. Quantitative results are shown in the figure on the *right* (*n* = 3). *J*, HK2 cells overexpressing Flag-tagged wild-type (WT) TFRC or K374R mutant TFRC were treated with or without acetic acid. Total cell lysates were subjected to anti-Flag immunoprecipitation (IP), and precipitated protein complexes were analyzed by immunoblotting using the indicated antibodies. Relative quantification of Lac-K/Flag signal intensity is presented in the *right panel* (n = 3). *K*, HK2 cells expressing wild-type TFRC or K374R mutant TFRC were cultured with lactate and treated with cycloheximide (CHX) for 0, 1, 3, and 6 h to block new protein synthesis. Total cell lysates were harvested at indicated time points and subjected to immunoblotting against TFRC and Tubulin. The line graph on the *right* shows quantitative analysis of TFRC/Tubulin relative expression across the CHX time course (n = 3). *L*, HK2 cells were transfected with Ad Flag-TFRC WT or Ad Flag-TFRC K374R, with or without lactate. CoIP was performed on total cell lysates using anti-Flag antibody, followed by Western blot analysis with designated antibodies. Quantitative results are shown in the figure on the *right* (n = 3).

We measured kidney lactate levels in *db/db* mice at 8, 16, 24, and 32 weeks of age, as well as in age-matched *db/m* mice. Results indicated that compared to *db/m* mice, *db/db* mice showed a significant rise of lactate levels in kidney starting at 16 weeks of age, with levels increasing with age (Fig. 1*A*). High levels of lactate can trigger lactylation modification (28, 29).We also assessed overall kidney lactylation modification levels in *db/db* mice and age-matched *db/m* mice, finding significantly higher levels in the *db/db* group (Fig. 1*B*).This suggests that a large number of lactylated proteins exist in the kidneys under a diabetic environment. Lactyl-proteome analysis of *db/db* kidney tissue identified lysine lactylation sites on multiple core ferroptosis regulatory proteins, including TFRC (K376), SLC3A2 (K42), GPX4 (K47), FTL1 (K144), TF (K315), ACO1 (K141, K379, K387, K396, K572, K615, K621, K639), GCLC (K492), GSS (K178, K186), and GSR (K278, K318, K324, K379, K448) (Table S1). Because TFRC is the rate-limiting transporter for iron uptake and a critical ferroptosis initiator, we selected TFRC K376 lactylation for subsequent mechanistic studies. Based on spectral data, the modified peptide LELSQNQNVKLIVK was detected at m/z 566.665 with a +3 charge state. The corresponding intact mass was calculated to be 1696.995 Da (566.665 × 3 − 3), whereas the theoretical mass of the unmodified peptide is 1624.951 Da. The mass difference of 72.044 Da is consistent with a mass shift characteristic of lysine lactylation. To identify the precise modification site among the two lysine residues within this peptide, we analyzed the MS/MS spectrum. The y_2_ ion corresponding to the C-terminal VK fragment was observed at m/z 246.17, matching the theoretical m/z of unmodified VK. This observation rules out lactylation on the C-terminal lysine and confirms that the modification occurs on the internal lysine residue. Similarly, in HK2 cells stimulated with lactate, the lactylation level of TFRC significantly increased (Fig. 1*D*). Sequence alignment of TFRC reveals not only that the homologous site is K374 in humans and K376 in mice, but also that this site is highly conserved across species, from yeast to humans (Fig. 1*E*).

We next investigated the lactylation levels of TRFC in the kidneys of *db/db* mice at distinct ages. These analyses revealed that kidney TFRC lactylation was significantly elevated with increasing age in *db/db* mice, alongside a similar significant upregulation of kidney TNF-α and KIM-1 expression in these mice (Fig. 1, *F*–*H*). To confirm whether diabetes modulates TFRC lactylation at the K374 residue, we generated a TFRC mutant in which the K374 residue was substituted with arginine (K374R)—a modification that abrogates lactylation at this site. Lactylation of the TFRC K374R mutant was markedly decreased relative to wild-type (WT) TFRC (Fig. 1*I*). Collectively, these data demonstrate that *db/db* mice display increased kidney TFRC lactylation specifically at the K374 residue. Collectively, these data demonstrate that kidney TFRC lactylation—specifically at the K376 residue—is increased in the kidneys of *db/db* mice.

To distinguish whether elevated TFRC lysine lactylation originates from lactate itself or merely extracellular acidification induced by lactate, HK2 cells expressing Flag-tagged wild-type TFRC (WT) or K374R mutant TFRC were treated with 10 mM sodium lactate or an equal concentration (10 mM) of acetic acid for 24 h. Immunoprecipitation with anti-Flag antibody followed by immunoblotting for pan-lactylation revealed that 10 mM lactate markedly boosted TFRC K374 lactylation, while equivalent acetic acid treatment did not alter TFRC lactylation levels (Fig. 1*J*). These data verified that the lactylation modification of TFRC is a lactate-specific event, independent of medium pH reduction.

We next verified that the TFRC K374R mutation does not inadvertently compromise TFRC protein stability or basal ligand-binding function, which could confound interpretation of its functional effects. First, we performed cycloheximide (CHX) chase assays under lactate stimulation to compare the degradation kinetics of Flag-TFRC-WT and Flag-TFRC-K374R (Fig. 1*K*). Time-course immunoblotting at 0, 1, 3, and 6 h after CHX treatment revealed nearly identical degradation curves and overlapping TFRC/Tubulin quantification profiles for the two constructs, with no detectable shift in protein half-life or steady-state abundance. Thus, the K→R substitution at position 374 does not alter TFRC protein stability or turnover. We next examined whether the K374R mutation disrupts the canonical interaction between TFRC and its ligand transferrin (TF) by co-immunoprecipitation (Fig. 1*L*). Under basal conditions, both WT and K374R TFRC could bind endogenous TF at comparable levels, indicating that the mutation does not impair the intrinsic ligand-binding interface. Upon lactate stimulation, however, WT TFRC exhibited robustly enhanced TF binding, whereas this lactate-induced increase was completely abolished in the K374R mutant.

Collectively, these data establish that the TFRC K374R mutant retains wild-type-like protein stability and baseline TF-binding capacity while selectively losing lactate-responsive upregulation of the TFRC-TF interaction. The functional defects observed in K374R-expressing cells can therefore be attributed specifically to the absence of K374 lactylation, rather than to nonspecific structural disruption of the mutant protein.

### TFRC K374R reduces ferroptosis in HK2 cells under lactate stimulation

**Figure 2:**
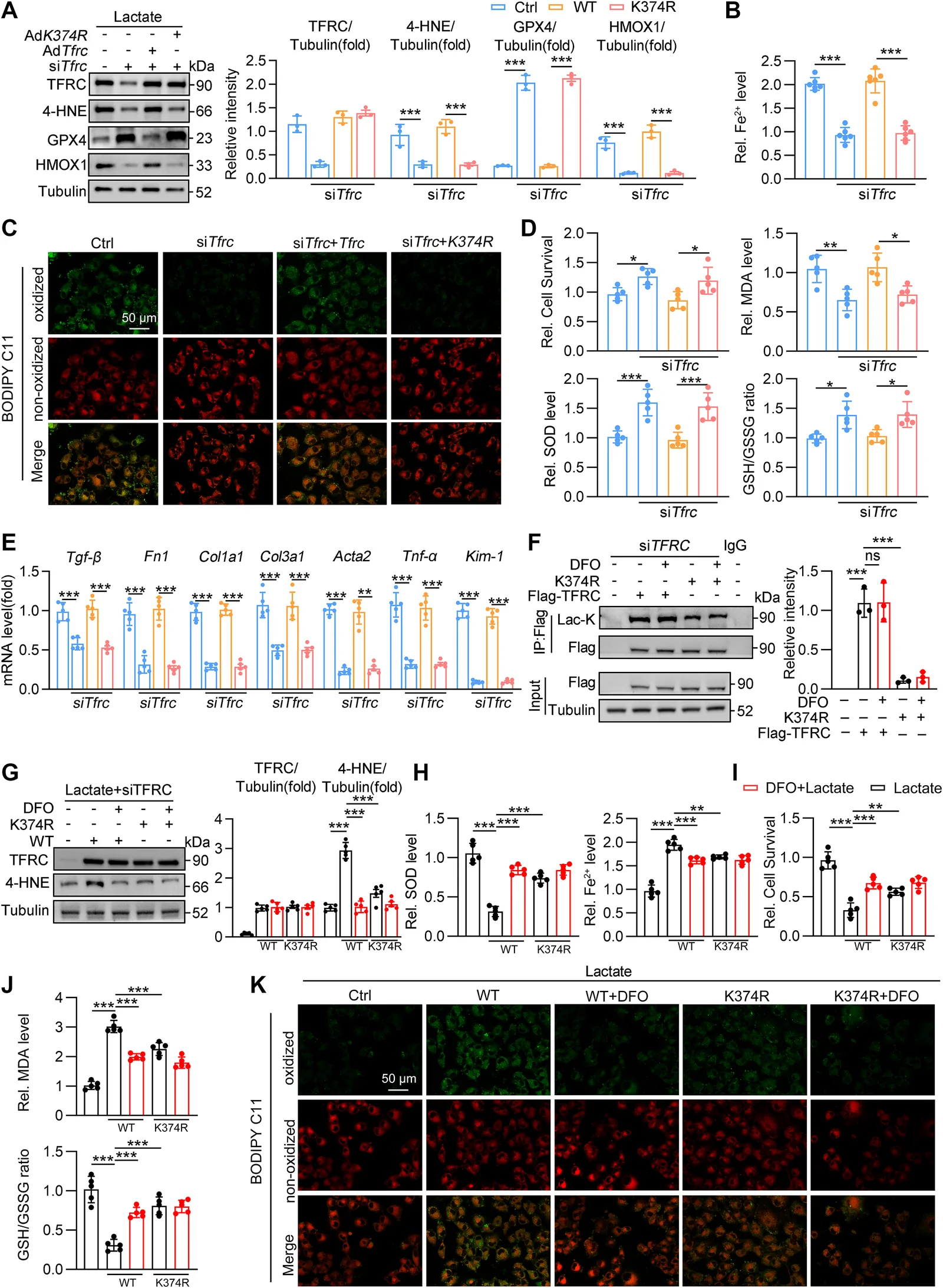
**TFRC K374R reduces ferroptosis in HK2 cells under lactate stimulation.***A–E*, HK2 cells were transfected with AdCtrl, Ad*Tfrc*, siCtrl, si*Tfrc*, or AdK374R in the presence of lactate. *A*: Total cell lysates were prepared and analyzed by Western blot using the indicated antibodies. Quantitative results are shown in the *right panel* (*n* = 3). *B*, a commercial assay kit was then used to detect Fe^2+^ levels in cell homogenates (*n* = 5). *C*, representative images of BODIPY 581/591 C11 staining. Scale bar: 50 μm. *D*, the cell survival, MDA, and GSH/GSSG ratio levels in cell homogenates were detected using a commercial kit (*n* = 5). *E*, the mRNA levels of *Tgf-β*, *Fn1*, *Col1a1*, *Col3a1*, *Acta2*, *Tnf-α*, and *Kim-1* were detected by qPCR in HK2s (*n* = 5). *F*, as shown in the figure, HK2 cells were transfected with si*Tfrc* in the presence of lactate, followed by transfection with either Ad-Flag TFRC WT or Ad-Flag TFRC K374R, with or without DFO (100 μM). CoIP was performed on total cell lysates using anti-Flag antibody, followed by Western blot analysis. Quantitative results are shown in the *right panel* (*n* = 3). *G*, the total cell lysates were prepared and subjected to western blotting using the indicated antibodie. The quantitative results of the Western blot are shown on the *right* (*n* = 5). *H*, a commercial assay kit measured Fe^2+^ levels and SOD in cell homogenates (*n* = 5). *I*: Cell survival in cell homogenates were measured using a commercial kit (*n* = 5). *J*, MDA, and GSH/GSSG ratio in cell homogenates were measured using a commercial kit (*n* = 5). *K*: Representative images of BODIPY 581/591 C11 staining. Scale bar: 50 μm.

Next, we examined whether this lactylation modification of TFRC affects ferroptosis in HK2 cells (a human proximal tubule epithelial cell line). We supplemented sodium lactate into the HK2 cell culture medium to simulate the lactate accumulation observed in the kidneys of diabetic mice. Our results found that the expression of GPX4 (a key ferroptosis suppressor), cell survival rate, SOD activity, and GSH/GSSG ratio (all of whitch indicators of antioxidant capacity against ferroptosis) were significantly increased. Meanwhile, the increase in 4-HNE and HMOX1 expression, MDA and Fe^2+^ accumulation as well as lipid peroxide levels accumulation (assessed *via* BODIPY C11 staining) induced by lactate stimulation was significantly attenuated by TFRC knockdown (Fig. 2, *A*–*D*). Notably, reintroducing wild-type (WT) TFRC reversed these changes mediated by TFRC deficiency, while the K374R mutant had no such effect (Fig. 2, *A*–*D*). Collectively, these results demonstrate that TFRC regulates ferroptosis in a lactylation-dependent manner, with the K374 site being critical for this regulatory function. Damage to kidney tubular epithelial cells is often accompanied by the expression of pro-fibrotic genes. For this reason, we detected kidney fibrosis markers (*Tgf-β*, *Fn1*, *Col1a1*, *Col1a3*, and *Acta2*) as well as kidney injury markers (*Tnf-α* and *Kim-1*). The results demonstrated that TFRC knockdown significantly reversed the lactate -induced upregulation of these markers (Fig. 2*E*). Furthermore, we found that in the presence of lactate, the TFRC K374R mutation significantly reduced the lactylation level of TFRC compared with WT TFRC (Fig. 2*F*). More importantly, when we treated the cells with the ferroptosis inhibitor DFO, we observed that DFO did not significantly affect the lactylation level both of WT or K374R TFRC (Fig. 2*F*), which suggests that lactate-driven TFRC lactylation is independent of ferroptosis. Collectively, these data highlight the critical role of the K374 residue in regulating the functional activity of TFRC: specifically, WT TFRC relies on the K374 site to mediate the lactate-induced upregulation of iron overload and kidney injury markers, whereas the K374R mutation abrogates this functional capacity of TFRC. Under lactate stimulation, DFO or TFRC K374R transfection increased cell survival, and the GSH/GSSG ratio in HK2 cells compared to TFRC-transfected cells. DFO or TFRC K374R also suppressed 4-HNE expression, and reduced MDA and Fe2+ accumulation, as well as lipid peroxide levels (Fig. 2, *G*–*K*). However, DFO intervention and TFRC K374R transfection showed no combined effect in reversing ferroptosis in HK2 cells (Fig. 2, *G*–*K*). These findings collectively demonstrate that the K374 residue of TFRC is critical for its role in mediating ferroptosis-associated kidney injury.

### The TFRC K374R mutation reduces ferroptosis and fibrosis in the kidneys of *db/db* mice

**Figure 3:**
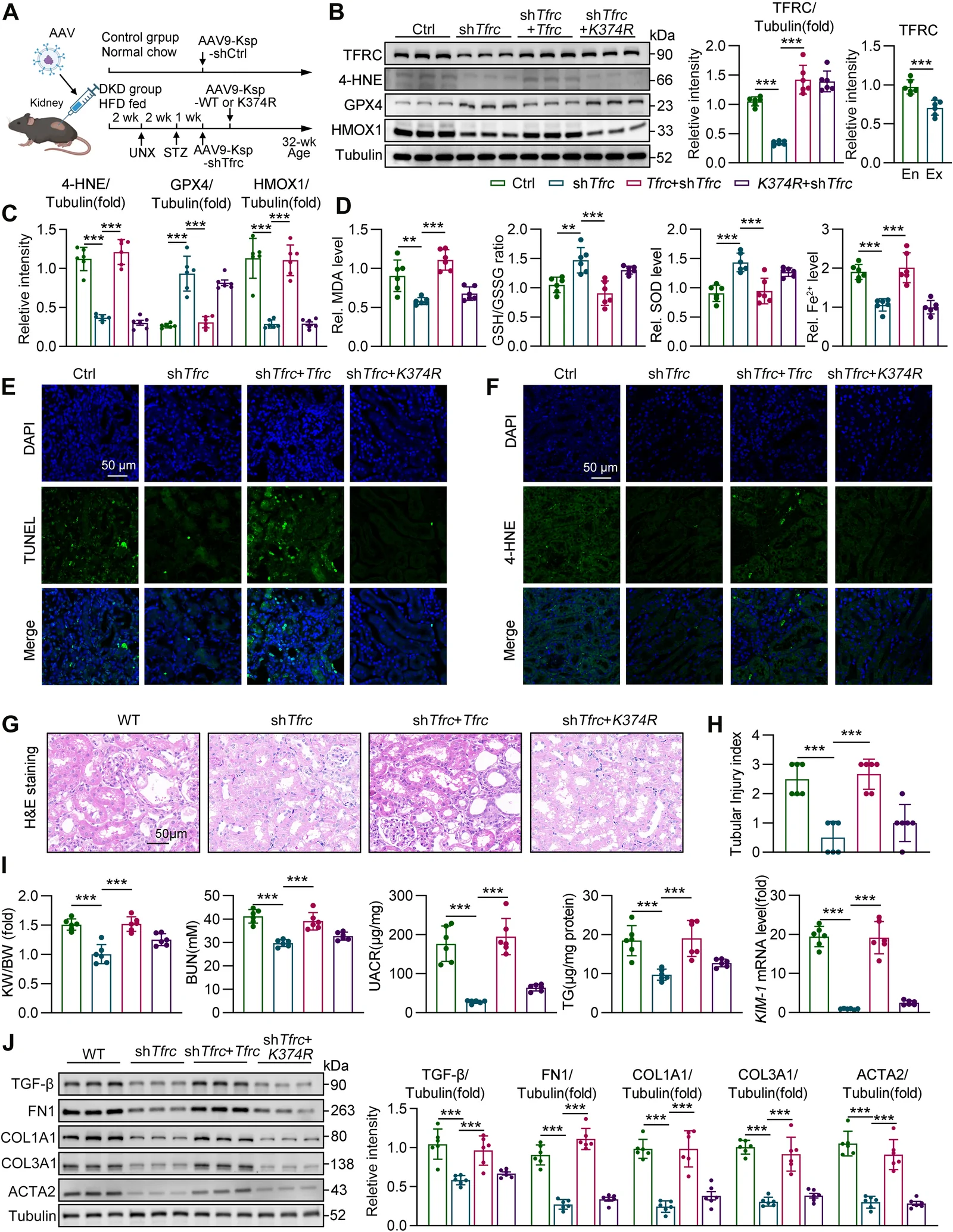
**TFRC K374R reduces Fe^2+^ induced cell death and fibrosis in *db/db* mouse kidneys.***A*, as shown in the figure, *db/db* mice were injected with AAV9-Ksp-shRNA targeting *Tfrc* (sh*Tfrc*), AAV9-Ksp-WT *Tfrc*, and AAV9-Ksp-K374R *Tfrc*. *B*, Western blot analysis and quantitative statistics of TFRC, 4-HNE, GPX4 and HMOX1 in renal tissues from each group (*n* = 6). Relative expression of endogenous (En) and exogenous (Ex) TFRC was quantified on the *right*. *C*, quantitative analysis of relative protein levels of 4-HNE, GPX4 and HMOX1 normalized to Tubulin (*n* = 6). *D*, the MDA, GSH/GSSG ratio, SOD, and Fe^2+^ levels in cell homogenates were detected using a commercial kit (*n* = 6). *E*, representative images of TUNEL staining in kidneys from mice. Scale bar: 50 μm. *F*: Representative images of 4-HNE staining in kidneys from mice. Scale bar: 50 μm. *G*, the quantitative result of H&E staining (*n* = 6). *H*, statistical quantification of tubular injury index (*n* = 6). *I*: The ratio of kidney weight to body weight, BUN, UACR, and TG of mice (*n* = 6). The mRNA level of *KIM1* was detected by qPCR (*n* = 6). *J*, the total cell lysates were prepared and subjected to Western blotting using the indicated antibodies. The quantitative results of the Western blot are shown on the right (*n* = 6).

To explore the role of K374 lactylation modification of TFRC in DKD pathogenesis, *db/db* mice were treated with AAV9-Ksp-shRNA targeting *Tfrc* (sh*Tfrc*) to knockdown endogenous TFRC. Meanwhile, two rescue groups were established by co-delivering sh*Tfrc* with either AAV9-Ksp-WT *Tfrc* (resistant to shRNA) or AAV9-Ksp-K374R *Tfrc* (Fig. 3*A*). By comparing these groups with the sh*Tfrc* group, we clarified the functional significance of TFRC K374 lactylation in kidney injury during DKD. We further quantified the relative protein abundance of endogenous (En) and exogenous (Ex) TFRC in mouse renal tissue. The relative intensity of endogenous TFRC protein was higher than that of exogenous AAV-delivered TFRC. Compared with the sh*Tfrc* group, overexpression of WT *Tfrc* completely abrogated the kidney protective effect induced by *Tfrc* knockdown. Specifically, the sh*Tfrc* + WT *Tfrc* group exhibited significantly elevated TUNEL positivity, 4-HNE levels, MDA content, and Fe^2+^ accumulation, along with upregulated ferroptosis-related markers in kidney tissues (Fig. 3, *B*–*F*). Concomitantly, the GSH–GSSG ratio and SOD activity were significantly reduced in this group compared to the sh*Tfrc* group. HE staining further confirmed more severe kidney tubular damage in the sh*Tfrc* + WT *Tfrc* group relative to the sh*Tfrc* group (Fig. 3, *D*–*G*). Additionally, the sh*Tfrc* + WT *Tfrc* group showed remarkably higher KW/BW ratio, BUN level, UCAR level, TG level, and *KIM-1* mRNA expression compared to the sh*Tfrc* group (Fig. 3*I*).

In sharp contrast, overexpression of K374R-mutated TFRC (sh*Tfrc* + K374R *Tfrc* group) failed to reverse the kidney protective effect of *Tfrc* knockdown. No significant differences were observed in the above-mentioned kidney injury indicators (KW/BW, BUN, UCAR, TG, *KIM-1* mRNA level) between the sh*Tfrc* + K374R *Tfrc* group and the sh*Tfrc* group (Fig. 3*I*), indicating that the K374 lactylation-deficient TFRC could not counteract the protective effect of *Tfrc* silencing. Consistent trends were observed in kidney fibrosis-related markers. Compared with the sh*Tfrc* group, overexpression of WT *Tfrc* (sh*Tfrc* + WT *Tfrc* group) significantly upregulated the mRNA levels of *Tgf-β*, *Fn1*, *Col1a1*, *Col1a3*, and *Acta2* (Fig. 3*J*). In contrast, overexpression of K374R-mutated TFRC (sh*Tfrc* + K374R *Tfrc* group) had no significant impact on the expression of these fibrosis markers compared to the sh*Tfrc* group, thus maintaining the antifibrotic effect mediated by *Tfrc* knockdown (Fig. 3*J*).

Collectively, these results demonstrate that the protective effects of *Tfrc* knockdown in *db/db* mice are recapitulated by the lack of TFRC K374 lactylation modification. Specifically, WT TFRC reverses the protective effects of sh*Tfrc*, while K374R-mutated TFRC fails to do so, thereby effectively suppressing kidney ferroptosis and fibrosis progression. These findings highlight TFRC K374 lactylation as a key functional modification that mediates the pro-kidney injury role of TFRC in DKD.

### Identification of p300 as the lactylase for TFRC K374

**Figure 4:**
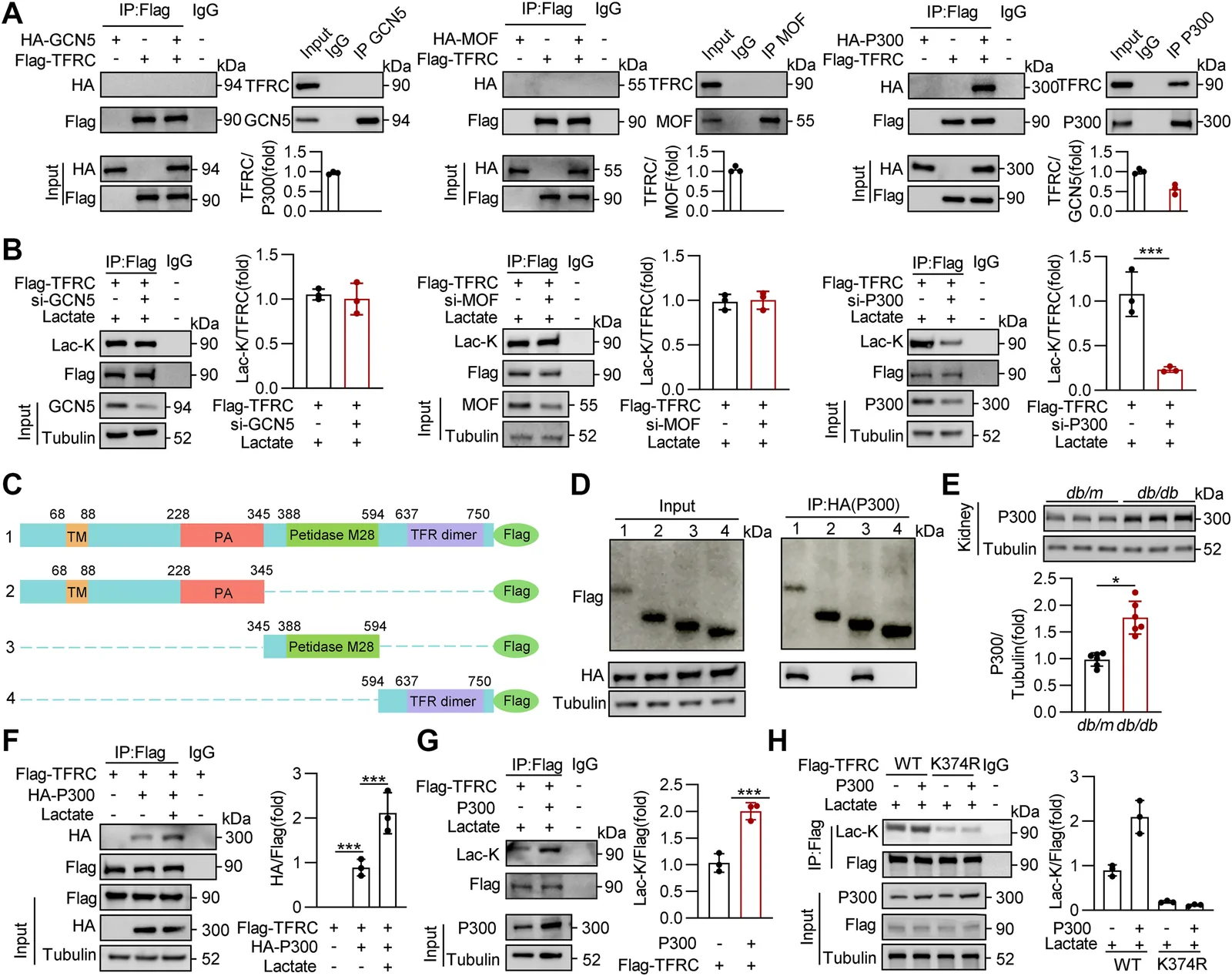
**The p300 levels were significantly elevated in *db/db* mice. It catalyzed the lactylation of TFRC at K374.***A*, *Left*: WT HK2 cells were transfected with Ad HA-GCN5/MOF/p300 and Ad-FLAG TFRC as shown. Total cell lysates underwent CoIP with anti-FLAG antibody and Western blot analysis with designated antibodies. *Right*: Total cell lysates from WT HK2 cells were CoIP with anti-GCN5/MOF/p300 antibodies and analyzed by Western blot. Quantitative results are shown in the *lower right panel* (*n* = 3). *B*, WT HK2 cells were transfected with FLAG-TFRC or siGCN5/MOF/p300 in the presence of lactate. CoIP was performed on total cell lysates using anti-FLAG antibody, followed by Western blot analysis with specific antibodies. Quantitative results are shown in the *right panel* (*n* = 3). *C*, schematic of TRIM65 deletion mutants. The numbers indicate the amino acid positions. *D*, FLAG-TFRC deletion mutants were coexpressed with HA-p300 in HK2 cells. *E*, Western blotting was performed on kidney cell lysates from *db/m* and *db/db* mice using anti-p300 antibodies. Quantitative results are shown below (*n* = 6). *F*, as shown in the figure, WT HK2 cells were transfected with Ad HA-p300 and FLAG-TFRC with or without 4 μM lactate. CoIP was performed on total cell lysates using anti-Flag antibody, followed by Western blot analysis. Quantitative results are shown in the *right panel* (*n* = 3). *G*, WT HK2 cells were transfected with Ad p300 and FLAG-TFRC with 4 μM lactate. CoIP was performed on total cell lysates using anti-Flag antibody, followed by Western blot analysis with the designated antibody. Quantitative results are shown in the *right panel* (*n* = 3). *H*, as shown in the figure, WT HK2 cells transfected with Ad p300, FLAG-TFRC, and FLAG-K374R were stimulated with lactate. CoIP was performed on total cell lysates using anti-Flag antibody, followed by Western blot analysis with specific antibodies. Quantitative results of Western blots are presented in the *right panel* (*n* = 3).

Next, we identified the enzyme(s) mediating the lactylation modification of TFRC at the K374 residue. Enzymes GCN5, MOF, and p300, which are histone acetyltransferases (HATs), can catalyze protein lactylation (30, 31). We first checked whether if GCN5, MOF, and p300 interact with TFRC. Both endogenous and exogenous Co-IP experiments showed that only p300 interacts with TFRC (Fig. 4*A*). We found that *p300* knockdown significantly reduced TFRC K374 lactylation, which suggests p300 is the primary enzyme responsible for this modification (Fig. 4*B*). In contrast, GCN5 and MOF knockdown did not affect lactylation at the TFRC K374 site (Fig. 4*B*). To identify the TFRC regions involved in TFRC-p300 binding, we used TFRC deletion mutants (Fig. 4*C*). Deleting the N-terminal or C-terminal domain had no effect on TFRC-p300 interaction. However, removing the Petidase M28 domain disrupted the interaction. Thus, this region is essential and sufficient for p300 binding (Fig. 4*D*). Subsequently, we assessed p300 expression in kidneys of *db/db* mice and observed a notable increase in its expression levels under diabetic conditions (Fig. 4*E*). Lactate stimulation intensifies the interaction between TFRC and p300 (Fig. 4*F*). Moreover, when HK2 exposed to lactate stimulation, p300 overexpression significantly promotes lactate-induced lactylation at TFRC K374 site on TFRC in these cells (Fig. 4*G*). When HK2 cells were transfected with Flag-TFRC WT or Flag-TFRC K374R, we observed that the TFRC K374R mutant abrogated the p300 overexpression-induced elevation in TFRC K374 lactylation (Fig. 4*H*). In summary, p300 drives the lactylation of TFRC at K374 and is markedly elevated in *db/db* mice.

### Inhibition of p300-mediated TFRC K374 lactylation to suppress lactate-induced ferroptosis in HK2 cells

**Figure 5:**
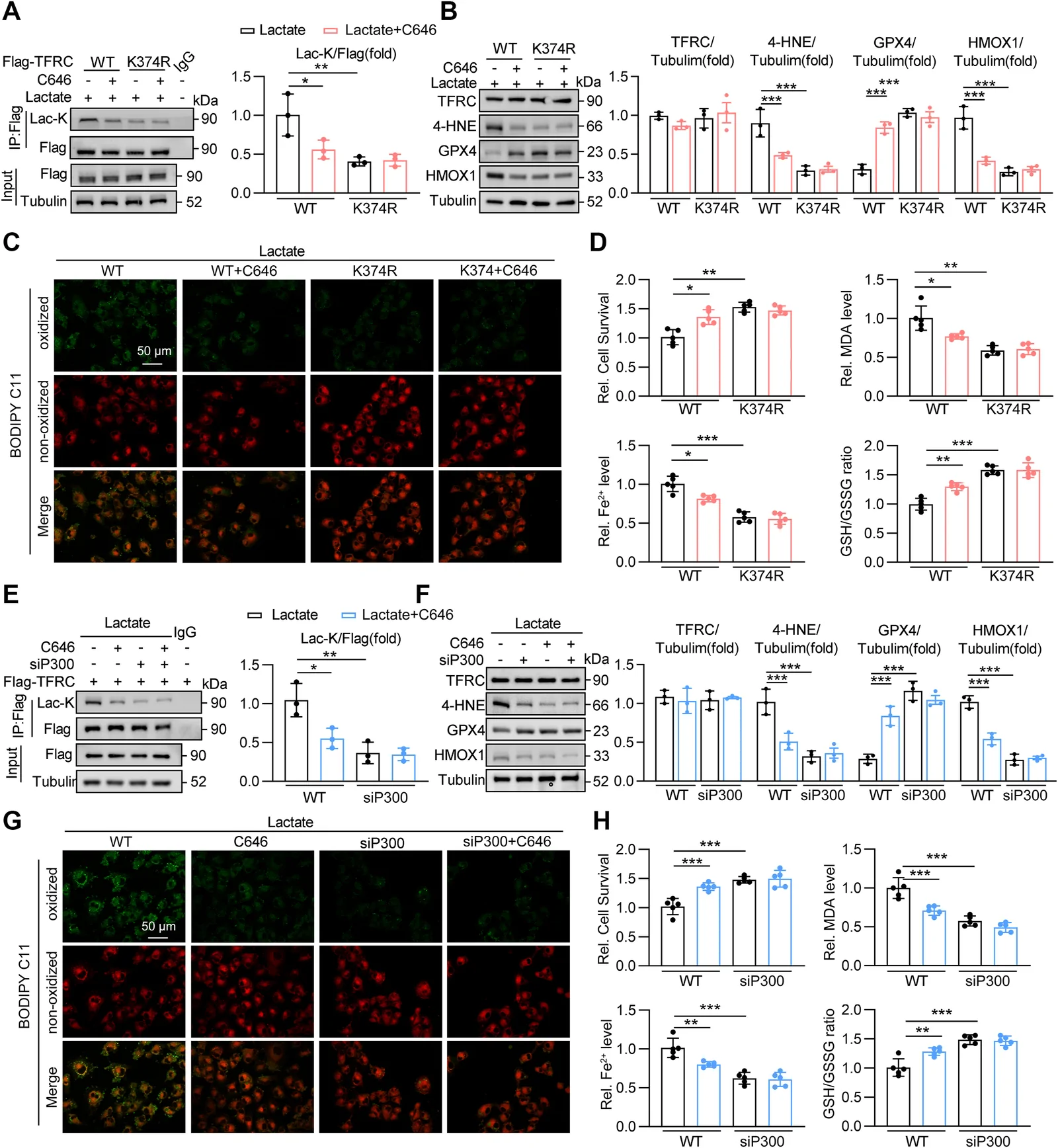
**The p300 inhibitor C646 alleviates lactate-induced ferroptosis.***A–D*, as shown in the figure, WT HK2 cells transfected with FLAG-TFRC and FLAG-K374R were treated with or without C646 (15 μM) in the presence of lactate. *A*: CoIP was performed using anti-Flag antibody on total cell lysates, followed by Western blot analysis with the designated antibody. Quantitative results from Western blots are shown in the figure on the right (*n* = 3). *B*: Perform Western blotting using the specified antibody. Quantitative results from the Western blot are shown in the figure on the *right*. (*n* = 3) *C*, representative images of BODIPY 581/591 C11 staining. Scale bar: 50 μm. *D*:The GSH/GSSG ratio, MDA, SOD, and Fe^2+^ levels in cell homogenates were detected using a commercial kit (*n* = 6). *E–H*, under lactate stimulation, WT HK2 cells transfected with FLAG-TFRC, FLAG-K374R, sip300, or siCtrl were treated with or without C646. *E*: CoIP was performed on the total cell lysate using anti-FLAG antibodies, and Western blotting was carried out using the specified antibodies. The quantitative results by Western blotting are shown in the *right* figure (*n* = 3). *F*, perform Western blotting using the specified antibody. Quantitative results from the Western blot are shown in the figure on the *right*. (*n* = 3), *G*, representative images of BODIPY 581/591 C11 staining. Scale bar: 50 μm. *H*, the GSH/GSSG ratio, MDA, SOD, and Fe^2+^ levels in cell homogenates were detected using a commercial kit (*n* = 6).

TFRC K374 plays a crucial role in ferroptosis, while C646 (a well-known p300 inhibitor) acts as an inhibitor of p300 (32, 33). We hypothesize that C646 intervention can reduce ferroptosis in HK2 cells. The experimental results indicate that C646 intervention significantly reduced the lactylation level of TFRC WT; however, it had no such effect on the lactylation level of TFRC K374R (Fig. 5*A*). Under lactate stimulation, C646 treatment reduced the expression of 4-HNE and HMOX1 as well as the accumulation of MDA, Fe^2+^, and lipid peroxide levels (Fig. 5, *B*–*D*). Meanwhile, C646 increased GPX4 expression, cell survival rate, and the GSH/GSSG ratio (Fig. 5*D*). However, in HK2 cells transfected with TFRC K374R, C646 intervention exerted no significant effect on these ferroptosis-related indices. To confirm whether C646 reduces ferroptosis and protects cells *via* inhibiting p300, we transfected siRNA *p300*(si*p300*) into HK2 cells and applied C646 treatment. Our results demonstrate that *p300* knockdown, C646 treatment, and combined sip300 and C646 treatment all reduced lactate-induced lactylation levels of TFRC (Fig. 5*E*). These treatments also promoted GPX4 expression, increased cell survival rates, elevated the GSH–GSSG ratio, suppressed 4-HNE and HMOX1 expression, and decreased levels of MDA, Fe^2+^, and lipid peroxides (Fig. 5, *F*–*H*). These results thus confirm that, under lactate stimulation, p300 exacerbates ferroptosis by promoting lactylation of TFRC at the K374 residue—an effect abrogated by C646 or p300 knockdown—and that this K374 lactylation is indispensable for p300’s pro-ferroptotic role.

### Screen-identified lysicamine (LCA) as a p300 inhibitor suppresses lactate-induced ferroptosis and kidney injury in *db/db* mice

**Figure 6:**
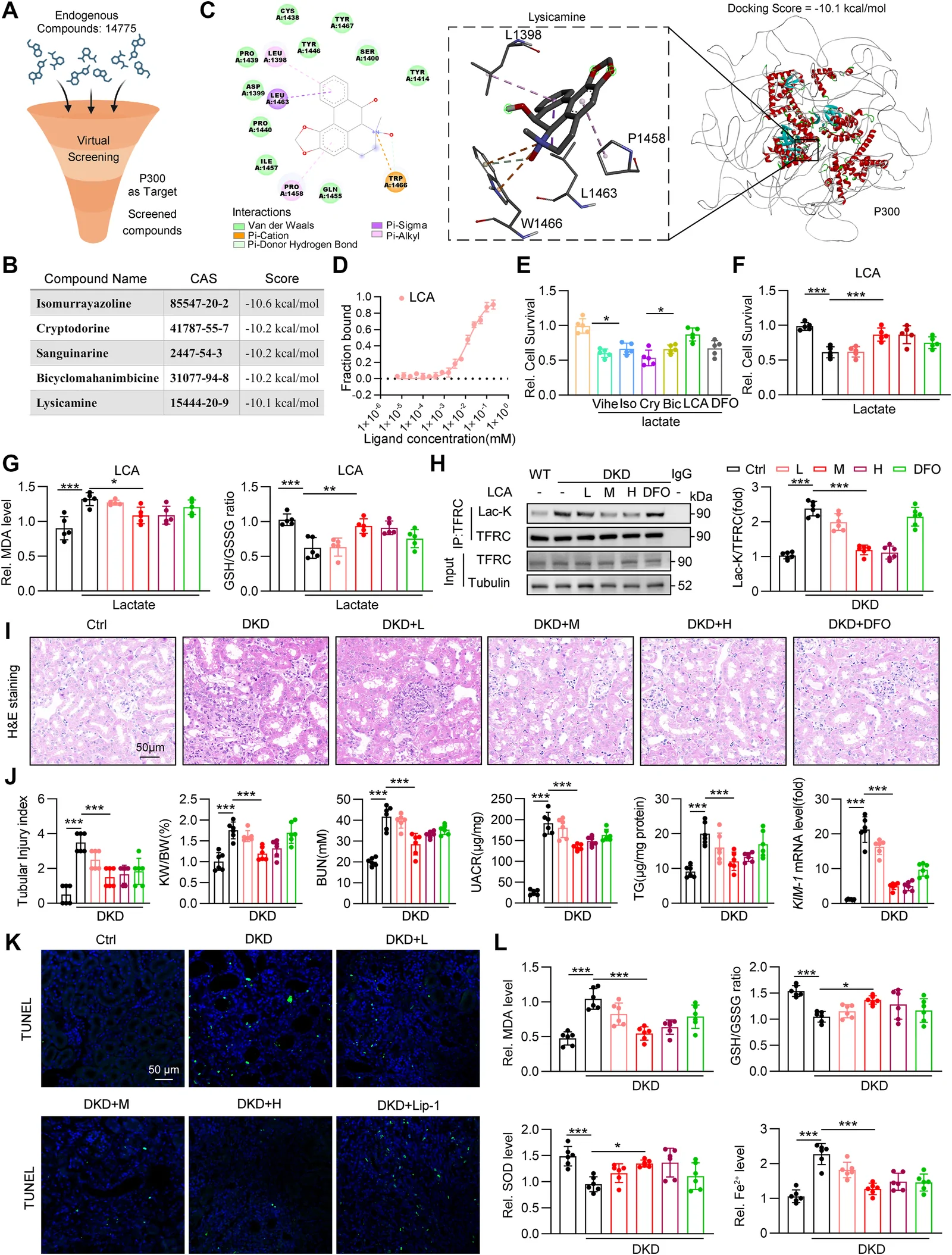
**The p300 inhibitor LCA alleviated kidney injury in *db/db* mice.***A*, schematic diagram of the structure-based virtual screening workflow. A library of 14,775 Endogenous compounds was screened using p300 as the target protein. *B*, chemical structures and docking scores of the top five candidate compounds. *C*, binding mode of Lifitegrast. *Right*: Overall ribbon structure of target protein, the ligand binding pocket is highlighted by *dashed rectangular box*; *Middle*: Three-dimensional spatial conformation of Lifitegrast embedded in protein binding cavity, key interacting amino acid residues are marked; *Left*: Two-dimensional interaction diagram displaying diverse non-covalent forces between Lifitegrast and pocket residues. The docking affinity score of Lifitegrast is −10.1 kcal/mol. *D*: Microscale thermophoresis (MST) assay measuring the binding affinity between LCA and recombinant p300 protein. *E* and *F*, relative cell viability of lactate-stimulated HK2 cells treated with gradient concentrations of LCA, Iso, Cry, BIC, DFO or lysicamine alone (*n* = 3). *G*, quantitative analysis of relative MDA level and GSH/GSSG ratio in lactate-treated HK2 cells with LCA intervention (*n* = 3). *H*, total cell lysates were subjected to CoIP with anti-FLAG antibody and western blotting using indicated antibodies. The quantitative results are shown in the figure on the *right* (*n* = 6). *I*, representative images of H&E staining in kidneys from mice (scale bar: 50 μm). *J*, quantification of tubular injury index, KW/BW ratio, BUN, UACR, urinary Tg and renal KIM-1 mRNA expression across groups (*n* = 6). *K*, representative images of TUNEL staining in kidneys from mice. Scale bar: 50 μm. *L*, quantitative detection of renal MDA, GSH/GSSG ratio, SOD activity and Fe^2+^ levels in DKD mice receiving different treatments (*n* = 6).

To identify novel potential regulators of ferroptosis, we performed virtual screening from an endogenous compound library (Fig. 6*A*). The top five candidate compounds identified *in silico* were subsequently validated using *in vitro* cellular assays (Fig. 6*B*). Of these candidates, LCA was prioritized based on its favorable docking score, and we directly verified its interaction with p300 by microscale thermophoresis (MST). The MST binding curve yielded a dissociation constant (Kd) of 14.6 μM, confirming that LCA specifically binds to p300 (Fig. 6*E*). Among these candidates, LCA exhibited a docking score of −10.1 kcal/mol (Fig. 6*C*). Functional screening revealed that only LCA significantly attenuated lactate-induced cell death (Fig. 6*D*), supporting its role as a candidate inhibitor of ferroptosis. Accordingly, we established an *in vitro* model of diabetic kidney lactate accumulation by supplementing HK2 cell culture medium with sodium lactate. Our results demonstrated that LCA treatment significantly improved cell viability, reduced the content of MDA, and elevated the GSH/GSSG ratio—all established indices of anti-ferroptotic and antioxidant capacity (Fig. 6, *F* and *G*), with medium and high concentrations exerting the most pronounced inhibitory effects.

To verify whether LCA acts specifically through p300, we examined the effects of LCA in p300-depleted cells. p300 knockdown by siRNA markedly reduced TFRC K374 lactylation, and LCA treatment produced no further suppression, with lactylation levels comparable between the sip300 and sip300 + LCA groups (Fig. S1*A*). Consistently, LCA reversed lactate-induced ferroptosis in control cells, as evidenced by reduced 4-HNE and HMOX1 and restored GPX4, yet these protective effects were completely abolished upon p300 silencing (Fig. S1*B*). These data confirm that LCA suppresses TFRC lactylation and ferroptosis exclusively through p300 inhibition p300.

We further explored the effects of different doses of LCA (low: 1 mg/kg, medium: 5 mg/kg, high: 25 mg/kg) and DFO (serving as a positive control) on kidney injury in *db/db* mice. Our results showed that both LCA (across doses), but not DFO, reduced TFRC K374 lactylation levels in the kidneys of *db/db* mice, with the medium-dose LCA group exerting the most significant inhibitory effect on this modification (Fig. 6*H*). HE staining revealed that medium-dose LCA intervention alleviated kidney tubular injury in *db/db* mice (Fig. 6, *I* and *J*). Consistently, medium-dose LCA administration decreased KW/BW ratio, BUN, UCAR, *KIM-1* mRNA levels and the level of cell apoptosis (assessed by TUNEL staining) (Fig. 6, *J* and *K*), collectively indicating that medium-dose LCA effectively mitigates kidney damage in *db/db* mice. In parallel, medium-dose LCA significantly reduced kidney MDA and Fe^2+^ levels (Fig. 6*L*) while increasing GSH–GSSG ratio and SOD activity in *db/db* mouse kidneys (Fig. 6*L*). Notably, medium-dose LCA had no adverse impact on kidney function in healthy mice, suggesting a favorable safety profile with no significant toxic side effects (data not shown). In summary, both the p300 inhibitor LCA and the ferroptosis inhibitor DFO confer kidney protection in *db/db* mice, with medium-dose LCA exhibiting the most pronounced efficacy.

We performed longitudinal monitoring of fasting blood glucose (FBG), body weight and blood pressure during the entire 11-weeks intervention, and started FBG and body weight quantification at week 10. Relative to normoglycemic db/m littermates, vehicle- and LCA-treated db/db mice both displayed persistent severe hyperglycemia, with indistinguishable FBG concentrations between the two diabetic subgroups (Fig. S1*C*). As a typical trait of the *db/db* strain, progressive weight gain was observed in all diabetic mice, and LCA intervention did not alter their weight growth profiles *versus* vehicle administration (Fig. S1*D*). Systolic blood pressure, diastolic blood pressure and mean arterial pressure were similar across db/m, vehicle-treated *db/db* and LCA-treated *db/db* groups (Fig. S1*E*). Collectively, LCA exerted no modulatory effects on blood glucose, body weight or blood pressure in *db/db* mice, which excludes indirect renal protection arising from improved systemic metabolism or hemodynamics. These data demonstrate that LCA ameliorates DKD *via* direct intrinsic protective effects within renal tubules.

## Discussion

Current research on TFRC regulation mainly focuses on its ubiquitination. For example, TRIB2 can tag TFRC for ubiquitination through βTrCP (34). This process reduces unstable intracellular iron levels and influences how sensitive hepatocellular carcinoma cells are to ferroptosis (34). TRIM33 increases liver cancer cells' susceptibility to ferroptosis by promoting TFRC ubiquitination. Targeting TFRC ubiquitination may be a potential therapeutic strategy for liver cancer (35).This study innovatively reveals that lactylation modification of TFRC plays a key role in diabetic kidney disease (DKD). Lactylation at TFRC's K374 site (K376 in mice) is significantly elevated in DKD mouse kidneys and is closely linked to increased levels of iron death, kidney fibrosis, and injury markers.This suggests that lactylation modification of TFRC may act as a novel regulatory mechanism in DKD, causing kidney injury and fibrosis, distinct from the previously identified ubiquitin-mediated regulatory pathway.

Ferroptosis is iron-dependent programmed cell death, crucial for various diseases' development. Empagliflozin may suppress ferroptosis by activating the AMPK-mediated NRF2 pathway (36, 37).Additionally, PHGDH eases DKD by regulating YB1/SLC7A11-mediated ferroptosis in podocytes (38). Similarly, Estrogen, particularly 2-hydroxyestradiol (2OH-E2), combats ferroptosis by donating hydrogen atoms to lipid peroxyl radicals, acting as a free radical scavenger (39).These findings indicate that targeting ferroptosis may be an effective treatment for chronic kidney disease (CKD). Similarly, ferroptosis research shows great potential in the field of oncology. For instance, SLC2A1 is highly expressed in many tumor tissues and crucially regulates ferroptosis in lung adenocarcinoma. SLC2A1 inhibits ferroptosis in colorectal cancer cells by boosting GPX4 expression (40). These studies show that modulating ferroptosis can impact tumor cell proliferation and metastasis, providing new ideas for cancer treatment. This study reveals that TFRC K374 lactylation contributes to DKD development by regulating ferroptosis, offering a new therapeutic target for DKD. Overall, ferroptosis related research has broad potential in disease treatment.

The p300, a HAT, is key for regulating gene expression, cell differentiation, and proliferation (41). The p300's role in kidney function isn't fully understood. However, this study shows p300 is the key enzyme for TFRC's K374 Lactylation in the kidney and is elevated in DKD mice. This suggests p300 may drive inflammation and fibrosis in kidney disease. LCA, a p300 inhibitor, showed strong kidney-protective effects in the study by reducing TFRC K374 lactate levels, relieving lactate-induced ferroptosis, and lessening kidney injury. Additionally, LCA also has anti-inflammatory and anti-tumor potential. For example, it can improve neurological deficits in traumatic brain injury by preventing ferroptosis and reducing inflammation (42). LCA regulates osteoblast differentiation by reducing inflammasome activation (43). LCA triggers prostate cancer cell apoptosis *via* autophagy induced by the ER stress/DAPK3/Akt signaling pathway (43). LCA's protective effects in kidney diseases position it as a promising new nephroprotective agent, offering a novel treatment option for conditions like DKD.

## Experimental procedures

### Details of mouse strains, rearing conditions, and experimentation

The seven-week-old male C57BL/KSJ diabetic *db/db* and *db/m* mice were purchased from the Shanghai Model Organisms Center, Inc. These mice were maintained in SPF units of the Animal Center of Shenzhen People’s Hospital with a 12 h light cycle from 8 AM to 8 PM, 23 °C± 1 deg, 60 to 70% humidity. Mice were allowed to acclimatize to their housing environment for 7 days before the experiments. At the end of the experiments, all mice were anesthetized and euthanized in a CO_2_ chamber, followed by the collection of kidney tissues. All animals were randomized before treatment. Mice were treated in a blinded fashion as the drugs used for treating animals were prepared by researchers who did not carry out the treatments. No mice were excluded from the statistical analysis. Studies were performed in accordance with the German Animal Welfare Act and reporting follows the ARRIVE guidelines.

### AAV-transfected mouse kidneys

Eight-week-old C57BL/6J mice received *in situ* kidney injection with AAV9-Ctrl (control group), AAV9-*Ksp*-WT *Tfrc* (which is resistant to shRNA) or AAV9-*Ksp*-K374R *Tfrc* (*n* = 6). Adeno-associated virus type 9 constructs including the GV736 empty vector and TFRC were provided by WZ Biosciences Inc. (Shandong, China). After two weeks of AAV infection, the mice underwent the diabetic model establishment as previously described.

### Study approval

All animal care and experimental protocols for *in vivo* studies conformed to the Guide for the Care and Use of Laboratory Animals, published by the National Institutes of Health (NIH; NIH publication no: 85-23, revised 2011), were approved by the Animal Care Committees of the Peking University Shenzhen Hospital (No. 2025-371), and were performed in compliance with the ARRIVE guidelines.

### Quantification and statistical analysis

All data were generated from at least three independent experiments. Each value was presented as the mean ± SD. All raw data were initially subjected to a normal distribution and analysis by one-sample Kolmogorov-Smirnov (K-S) nonparametric test using SPSS 22.0 software. For animal and cellular experiments, a two-tailed unpaired student’s *t* test was performed to compare the two groups. One-way ANOVA followed by the Bonferroni’s *post hoc* test was used to compare more than two groups. To avoid bias, all statistical analyses were performed blindly. Statistical significance was indicated at ∗*p* < 0.05, ∗∗*p* < 0.01, and ∗∗∗*p* < 0.001.

### Cell culture and transfection

The human proximal tubular epithelial cell line (HK2 cells) (Cat. No. CRL-2190) were purchased from the American Type Culture Collection, which were STR authenticated by the supplier. Cells were cultured in Minimum Essential Medium (Thermo Fisher Scientific, Shanghai, China; Cat. No. 10373017). All kinds of media were supplemented with 10% FBS (Gibco), 100 U·mL−1 of penicillin (Gibco), and 100 mg•mL−1 of streptomycin (Gibco). All cells were cultured in a 37 °C incubator containing 5% CO2 and 95% air.Cells were regularly checked for *mycoplasma* in a standardized manner, by a qPCR test, performed under ISO17025 accreditation to ensure work was conducted in mycoplasma-negative cells.

Adenovirus particles used in the article were provided by WZ Biosciences Inc. Adenovirus was used to transfect HK2 cells for 6 h (adenovirus particles containing empty vectors were used as controls), and then replace the fresh medium and continue to culture. The commercial siRNA Ctrl, siRNA *Tfrc* were transfected using Lipofectamine 3000 (Invitrogen) according to the manufacturer’s instructions.

### Protein extraction and Western blot analysis

Protein extraction and Western blot analysis was performed according to the previous study (44). In brief, After treatment, the cells and mouse kidney tissues were homogenized in RIPA lysis buffer (Cat. No. R0010, Solarbio) supplemented with protease inhibitors (Cat. No. P6730, Solarbio) and PMSF (Cat. No. R0010, Solarbio). After being centrifuged for 5 min (10,000*g*) at 4 °C, the supernatant was collected, and the protein concentrations were quantified using Pierce BCA Protein Assay Kit (Cat. No. 23225, Thermo Fisher Scientific). Protein lysates were mixed with 5× protein loading buffer and boiled for 5 min at 95 °C. Protein samples were fractionated on 4 to 12% SDSPAGE gel, transferred to PVDF membranes, and blocked in blocking buffer (5% fresh dry fat-free milk, 0.1% Tween 20 in TBS). Membranes were incubated with primary antibodies (list provided below) overnight at 4 °C. Blots were developed using HRP-conjugated anti-mouse or anti-rabbit IgG secondary antibodies (1:10,000, Abcam) and WesternBright ECL HRP substrate (Advansta). Densitometry analysis was performed using Quantity One Software (Bio-Rad Laboratories), and the protein levels were normalized to Tubulin. The details of full length western blots were shown in Supplemental Material file.

### Histological analysis

Kidney tissue was embedded in paraffin and sliced into 5 μm thick serial sections using a paraffin slicer. For kidney histology, the paraffin sections were stained using Modified Hematoxylin-Eosin (HE) Stain Kit (Cat. No. G1121, Solarbio & Technology). Positive cells were morphometrically quantified with image processing software (ImageJ) by a technician who blinded to the experiments.

### Quantitative real-time PCR

QPCR was performed according to the previous study (45). In brief, Total RNAs were extracted by Trizol (Invitrogen) and then dissolved in an appropriate amount of RNase water. cDNA was obtained by a reverse transcription kit purchased from TransGen Biotech. qPCR was performed using the ABI StepOnePlusTM Real-time PCR system (Applied Biosystem) with specific primers (Supplemental Material). The relative mRNA levels of target genes were analyzed using the 2−ΔΔCT method. Tubulin was used as a housekeeping gene for analysis.

### Co-immunoprecipitation (Co-IP)

After treatment, the cells were lysed in an ice-cold co-IP buffer containing 20 mM Tris-HCl (pH 8.0), 100 mM NaCl, 1 mM EDTA, and 0.5% NP-40, supplemented with a protease inhibitor cocktail (Roche, 04693132001), for 30 min. The cell homogenates were then centrifuged at 13,000*g* for 15 min, and the resulting supernatant was incubated overnight at 4 °C on a shaker with primary antibodies (anti-Flag, anti- TFRC, anti-GCN5, anti-MOF, anti-HA, anti-Lac-K, anti-p300 or anti-IgG). To ensure complete saturation of the primary antibodies, sufficient cell lysates were cultured and collected for IP. The mixture of antibodies and proteins was subsequently incubated with protein A/G-agarose beads (Thermo Fisher Scientific, Cat. No. 78610) at 4 °C for 3 h. The beads were washed 5 to 6 times with cold IP buffer and resuspended in loading buffer. The cell lysates and immunoprecipitates were denatured in loading buffer at 95 °C for 5 min, and western blotting analysis was performed.

### Analysis of the conservation of lysine 374 in TFRC

A gene panel of the NCBI database (https://www.ncbi.nlm.nih.gov/gene/) was used to download amino acid sequences of proteins from multiple species and subsequently compare sequence conservation between species using the UGENE software (version 39; the software can be downloaded from the following website: http://ugene.net/).

### Immunofluorescent (IF) staining

IF was performed according to the previous study (46). In brief, Kidney tissues were embedded in paraffin and cut into 4-mm-thick sections. After blocking with goat serum for 30 min, the tissues were incubated with primary antibodies against anti-4-HNE (1:100, Novus, Bio-Techne China, Cat. No. MAB3249) at 4 °C overnight. Alexa Fluor 488 phalloidin (1:400, Cell Signaling Technology; Cat. No. 8878S) was included as secondary antibodies. As negative controls, the primary antibodies were exchanged for nonimmune serum from the same species. The samples were counterstained with DAPI at a dilution of 1:1000 for 15 min. All sections were analyzed using a Laica microscope.

### Ferroptosis analyses

The researchers employed several commercially available kits and probes, meticulously following the manufacturers’ instructions. The MDA kit (Beyotime, Cat. No. S0131S) was utilized to measure malondialdehyde (MDA) levels. Superoxide dismutase (SOD) activity was determined using the SOD kit (Nanjing Jiancheng Bioengineering Institute, Cat. No. A001-3-2). For detecting intracellular iron levels, the FerroOrange probe (Dojindo Cat. No. F374) was implemented. Lipid peroxidation was evaluated with the BODIPY 581/591 C11 probe (ThermoFisher Scientific, Cat. No. D3861). These kits are all commercially available products with clear instructions and are widely used in the relevant research field. We have strictly adhered to the manufacturers’ instructions during the experiment process and have also ensured that all necessary controls were in place.

### Iron content

The tissue iron assay kit (Nanjing Jiancheng, Cat. No. A039-2-1) was used for iron content measurement. In accordance with the protocol, the kidney tissues were homogenized in phosphate-buffered saline (PBS), followed by the addition of the iron color-developing agent. After the vortex, the mixture was clarified by centrifugation at 1500*g* for 10 min. Finally, the absorbance was determined at a wavelength of 520 nm.

## Data availability

All data generated or analyzed during this study are included in this published article and its supporting information files. Raw data is available upon request.

## Supporting information

This article contains supporting information.

## Conflict of interest

The authors declare that they have no conflicts of interest with the contents of this article.
