# Supplementary material for: Lysine-374 lactylation of TFRC is essential for TFRC-mediated kidney tubular ferroptosis in diabetic kidney disease
Source: J Biol Chem. 2026 Aug 10;302(10):113429. doi: 10.1016/j.jbc.2026.113429 (PMC13570337; doi:10.1016/j.jbc.2026.113429)
Supplement: Supplementary file 1 [file mmc1.docx]

**Lysine-374 Lactylation of TFRC Is Essential for TFRC-Mediated kidney Tubular Ferroptosis in Diabetic Kidney Disease**

**1. Supplementary Figure and Figure legend**

**Supplementary figure 1:** Lysicamine (LCA) inhibits p300-mediated TFRC lactylation and ferroptosis in lactate-treated HK2 cells. A: HK2 cells overexpressing Flag-TFRC were transfected with si-p300 or negative control siRNA, followed by lactate treatment with or without LCA. Total cell lysates were subjected to anti-Flag immunoprecipitation, and TFRC lactylation was detected via immunoblotting with anti-Lac-K antibody. The quantitative analysis of Lac-K/Flag signal intensity is shown on the right (*n* = 3). B: Western blot detection of TFRC, 4-HNE, GPX4 and HMOX1 in lactate-stimulated HK2 cells with or without si-p300 transfection and LCA treatment. Relative protein expression normalized to Tubulin was quantified (n = 3).

**Supplementary figure 2**: Lysicamine (LCA) has no obvious influence on blood glucose, body weight and blood pressure in db/db mice during 11-week intervention. A: Serial detection of fasting blood glucose (FBG) levels of db/m, db/db and db/db+LCA groups at each time point within 11 weeks (n = 6). B: Dynamic recording of body weight of three groups over the 11-week treatment period (n = 6). C: Statistical analysis of systolic arterial pressure (AP), diastolic AP and mean AP in db/m, db/db and db/db+LCA mice at the end of intervention (n = 6).

**2. Supplementary Table**

**Supplementary Table S1**

| Gene name | Position | Amino acid | Modified sequence |
| --- | --- | --- | --- |
| Slc3a2 | 42 | K | IK[Lac (K)]VAEDETEAGVK |
| Gpx4 | 47 | K | SMHEFSAK[Lac (K)]DIDGHMVCLDK |
| Ftl1 | 144 | K | K[Lac (K)]M[Oxidation (M)]GNHLTNLR |
| Tfrc | **376** | **K** | **LELSQNQNVK[Lac (K)]LIVK** |
| Tf | 315 | K | DLLFK[Lac (K)]DSAFGLLR |
| Aco1 | 141 | K | RADSLQK[Lac (K)]NQDLEFER |
| Aco1 | 379 | K | RPQDK[Lac (K)]VAVSEMK |
| Aco1 | 387 | K | K[Lac (K)]DFESCLGAK |
| Aco1 | 396 | K | DFESCLGAK[Lac (K)]QGFK |
| Aco1 | 572 | K | IDFEK[Lac (K)]EPLGVNAQGR |
| Aco1 | 615 | K | EVYQK[Lac (K)]IETVNK |
| Aco1 | 621 | K | IETVNK[Lac (K)]SWNALAAPSEK |
| Aco1 | 639 | K | LYAWNPK[Lac (K)]STYIK |
| Gclc | 492 | K | DICK[Lac (K)]GGNAVVDGCSK |
| Gss | 178 | K | EASK[Lac (K)]ILSNNPSK |
| Gss | 186 | K | ILSNNPSK[Lac (K)]GLALGIAK |
| Gsr | 278 | K | K[Lac (K)]TSSGLELQVVTSVPGR |
| Gsr | 318 | K | DPNSK[Lac (K)]GLNLNK |
| Gsr | 324 | K | GLNLNK[Lac (K)]VGIQTDEK |
| Gsr | 379 | K | LFECK[Lac (K)]QDSK |
| Gsr | 448 | K | MVCANK[Lac (K)]EEK |

**Supplementary Table S2**

| Sequence | LELSQNQNVKLIVK |
| --- | --- |
| Length | 14 |
| Modifications | Lac (K) |
| Modified sequence | _LELSQNQNVK[Lac (K)]LIVK_ |
| Protein accession | Q62351 |
| Protein description | Transferrin receptor protein 1 OS=Mus musculus OX=10090 GN=Tfrc PE=1 SV=1 |
| Gene name | Tfrc |
| MW [kDa] | 85.732 |
| Charge | 3 |
| Score | 45.52686 |
| Intensity DB_K1 | 55.72733 |
| Intensity DB_K2 | 14.69074 |
| Intensity DB_K3 | 25.00296 |
| Intensity DB_K4 | 27.88811 |

**Supplementary Table 3 Primers used in qPCR**

| Gene | Forward Primer (5’→3’) | Reverse Primer (5’→3’) |
| --- | --- | --- |
| *mTgf-β* | CTCCCGTGGCTTCTAGTGC | GCCTTAGTTTGGACAGGATCTG |
| *mFn1* | ATGTGGACCCCTCCTGATAGT | GCCCAGTGATTTCAGCAAAGG |
| *mCol1a1* | GCTCCTCTTAGGGGCCACT | CCACGTCTCACCATTGGGG |
| *mCol3a1* | CTGTAACATGGAAACTGGGGAAA | CCATAGCTGAACTGAAAACCACC |
| *mActa2* | CCCAACTGGGACCACATGG | TACATGCGGGGGACATTGAAG |
| *m**Tnf-α* | GACGTGGAACTGGCAGAAGAG | TTGGTGGTTTGTGAGTGTGAG |
| *mKim-1* | GTTAAACCAGAGATTCCCACACG | TCTCATGGGGACAAAATGTAGTG |

**Supplementary Table 4 Antibodies used in Western blottings**

| Antibodies | Source | Identifier | RRIDs |
| --- | --- | --- | --- |
| Lac-K | Thermo Fisher | PA5-116901 | AB_2901531 |
| Tubulin | proteintech | 11224-1-AP | AB_2210206 |
| TFRC | Thermo Fisher | 13-6800 | AB_2533029 |
| TNF-α | Thermo Fisher | MM350C | AB_223527 |
| KIM-1 | proteintech | 30948-1-AP | AB_3669790 |
| 4-HNE | Abcam | ab46545 |  |
| GPX4 | proteintech | 67763-1-Ig | AB_2909469 |
| HMOX1 | proteintech | 10701-1-AP | AB_2118685 |
| TGF-β | proteintech | 81746-2-RR | AB_3670503 |
| FN1 | proteintech | 15613-1-AP | AB_2105691 |
| COL1A1 | Santa Cruz biotechnology | sc-293182 | AB_2797597 |
| COL3A1 | Abcam | ab7778 | AB_306066 |
| ACTA2 | proteintech | 14395-1-AP | AB_2223009 |
| GCN5 | proteintech | 66575-1-Ig | AB_2881935 |
| MOF | proteintech | 13842-1-AP | AB_2146894 |
| TF | proteintech | 17435-1-AP | AB_2035023 |
| P300 | proteintech | 20695-1-AP | AB_3085614 |
